# Bioinspired Optical Sensor for Remote Measurement of Small Displacements at a Distance

**DOI:** 10.3390/biomimetics3040034

**Published:** 2018-10-30

**Authors:** Susan A. Frost, Leslie A. Yates, Hiroyuki S. Kumagai

**Affiliations:** 1 National Aeronautics and Space Administration (NASA) Ames Research Center, P.O. Box 1, M/S 269-3, Moffett Field, CA 94035, USA; 2 AerospaceComputing, Inc., 465 Fairchild Drive, Suite 224, Mountain View, CA 94043, USA; lyates@aerospacecomputing.com (L.A.Y.); hkumagai@aerospacecomputing.com (H.S.K.)

**Keywords:** bioinspired optical sensor, compound eye sensor, optical displacement sensor, remote displacement measurement, fault creep measurement, structural displacement measurement, quasi-Gaussian signal processing

## Abstract

Identifying appropriate sites for landing a spacecraft or building permanent structures is critical for extraterrestrial exploration. By tracking the movement of land masses and structures on a planetary surface, scientists can better predict issues that could affect the integrity of the site or structures. A lightweight, low-cost, low-power bioinspired optical sensor is being developed at the National Aeronautics and Space Administration (NASA) Ames Research Center to remotely measure small displacements of land masses on either side of a fault. This paper describes the sensor, which is inspired by the compound eye vision system found in many insects, and the algorithms developed to estimate displacement. The results are presented for indoor and outdoor tests using the sensor to measure the displacement of a specially designed target that is located 0.35, 6, and 30 m from the sensor and is moved 10 mm to the left and right of a centered position, simulating the displacement of land masses on either side of a fault. Measurement uncertainties estimates were a few tenths of a millimeter when the target was located 0.35 and 6 m from the sensor. At the 30 m distance, corrections were required to obtain accuracies in the order of 1 mm.

## 1. Introduction

Identifying appropriate sites for landing a spacecraft or building permanent structures is critical for extraterrestrial exploration, including asteroids, moons, and planets. By tracking the movement of land masses or structures on a planetary surface, scientists can better predict issues that could affect the integrity and safety of the site or the structures. A fault in the crust of a planetary body is a planar fracture or discontinuity in a volume of rock that experiences displacement due to movement of the rock. If a landing site or structure is located near a fault, scientists will need to monitor the movement of land on either side of the fault to assess the hazard of the fault. Environmental conditions on extraterrestrial surfaces are vastly different than conditions found on Earth, hence scientists and engineers will need to closely monitor structures built in these uncertain environments for movement that could signify support or structural issues. The National Aeronautics and Space Administration (NASA) has suggested that “advancements in additive manufacturing, or 3-D printing, may make it possible to use regolith harvested on the Moon, Mars and its moons, and asteroids to construct habitation elements on extraterrestrial surfaces, such as living quarters and storage facilities” [1]. Construction methods using raw materials from a planet being explored, also known as in situ resource utilization, are motivated, in part, by the high cost of transporting materials. Structures built with extraterrestrial materials not found on Earth would require close monitoring due to incomplete knowledge of the material properties. The sensor reported here could be used for these remote sensing applications since it is able to remotely measure small displacements (mm to cm) at long distances (1–60 m). Here, we focus on the application of monitoring fault movement, although the sensor and algorithms presented in this work could be used to remotely measure the displacement of many other objects.

**Figure 1:**
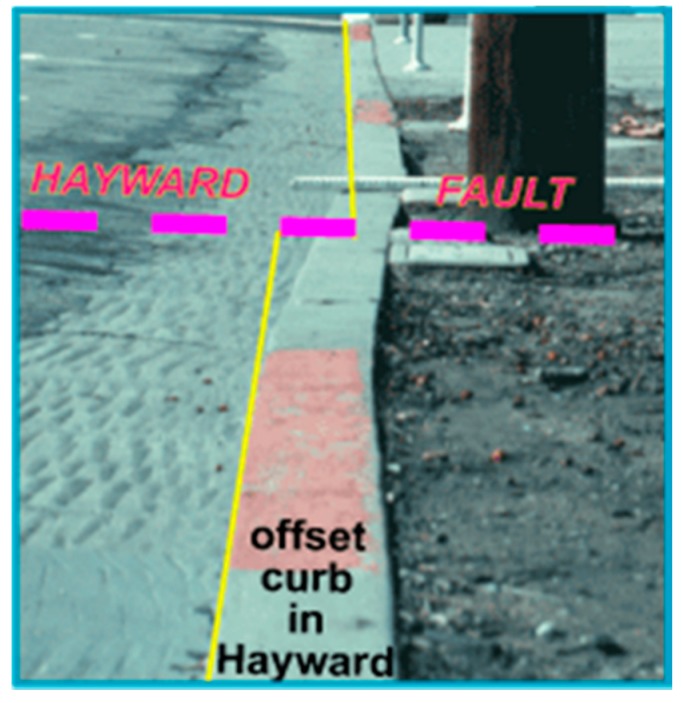
Evidence of fault creep in Hayward, CA, USA. Credit: U.S. Geological Survey (USGS) [3].

Faults in the Earth’s crust can move slowly without detectable tremors, a process called aseismic deformation or fault creep (Figure 1). The movement of some active faults is in the range of a few millimeters at a time, with a cumulative movement of a few centimeters a year. Fault creep can relieve strain on a fault, potentially reducing the hazard of a fault. Measurements of these small displacements at many locations along the fault are needed to accurately model inter-seismic and post-seismic fault creep and estimate fault hazard. The cost of current technology to measure earthquake fault creep limits deployment. In particular, one of the most promising fault creep meters uses an Invar wire (or a graphite rod) that is anchored to concrete monuments on either side of a fault to measure strain which is then converted to displacement [2]. The weight and installation requirements of this system make it impractical for applications on other planetary bodies, whereas the bioinspired sensor described here is a small fraction of the weight, making it a good alternative.

A novel low-cost, low-power bioinspired optical sensor, which could be deployed at many locations, is being developed at NASA Ames Research Center (NASA Ames) for measuring fault creep with funding from the United States Geological Survey Innovation Center for Earth Sciences, United States Department of the Interior. This sensor could play an important role in the monitoring of landslides (especially shear failures at landslide margins) and could help monitor volcanic deformation over short spatial scales. Additionally, the bioinspired sensor could be used in many other applications requiring displacement measurements, such as the movement of civil structures.

The bioinspired sensor for fault monitoring builds upon a bioinspired sensor developed for near real-time estimation of the position of an aircraft wing under load [4,5,6,7]. For the aircraft application, it was shown that the bioinspired sensor and associated tracking algorithms could outperform a traditional digital camera, where movement results in blurred images. Additional studies using a sensor with a similar design demonstrated the advantages of a compound eye-inspired sensor over a digital camera for processing certain types of moving targets [8]. Since the bioinspired sensor only has seven photodetectors, the power and bandwidth requirements for signal acquisition and data processing are much lower than for a standard digital camera.

Inspiration for NASA’s bioinspired sensor comes from the compound eye-based vision system found in many insects, including the common housefly (*Musca domestica*) [7,8,9,10]. The insect compound eye consists of many light sensitive cells (ommatidia) with overlapping fields of view. Each ommatidium gives a quasi-Gaussian response to visual stimuli [11]. These characteristics of the vision system along with neural superposition endow the insect with the ability to quickly detect very small movements of an object. The sensor design reported in this paper draws inspiration from the quasi-Gaussian response, multiple aperture, and overlapping fields of view found in the insect eye. It does not mimic the neural superposition properties or the arrangement of multiple photoreceptors within each ommatidium found in a neural superposition type compound eye.

**Figure 2:**
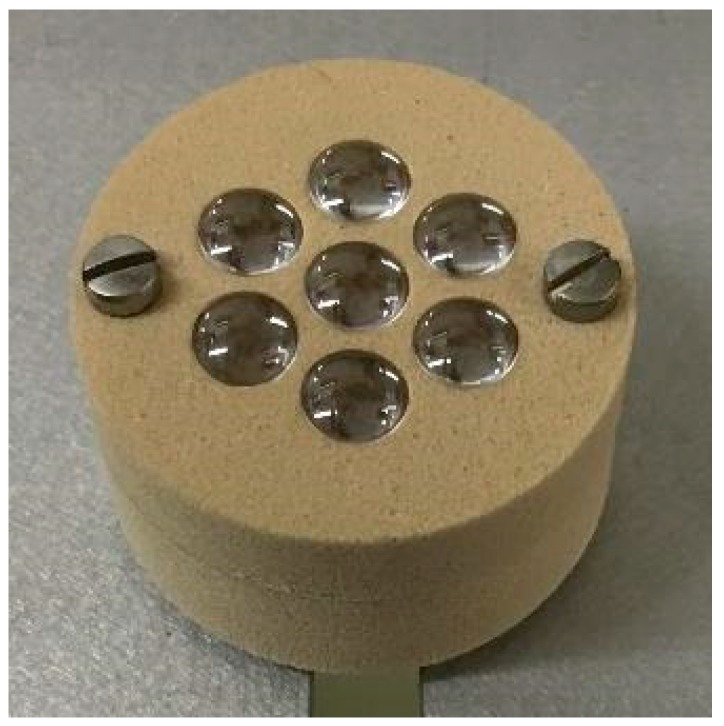
NASA Ames seven-lens sensor head (31.75 mm diameter) with 6 mm lenses.

The seven-lens compound eye-inspired sensor designed and built at NASA Ames has seven individual sensors with overlapping fields of view (Figure 2). Each individual sensor has a lens that focuses incoming light through an airgap onto the face of a plastic optical fiber (POF). The light passes through the POF to a photodetector on a custom printed circuit board (PCB) that was designed and built by researchers at the University of Wyoming, Laramie, WY, USA [7,9]. The PCB amplifies and converts the current coming from the photodetector to a voltage and performs filtering of the signals. The individual sensors, with airgaps that blur the image, and the electronics on the PCB, are designed to give the desired quasi-Gaussian response to stimuli. The overlap of the sensors’ fields of view is achieved through the arrangement of the sensors in the sensor head. The quasi-Gaussian response and the overlapping fields of view enable an accurate estimation of target movement.

**Figure 3:**
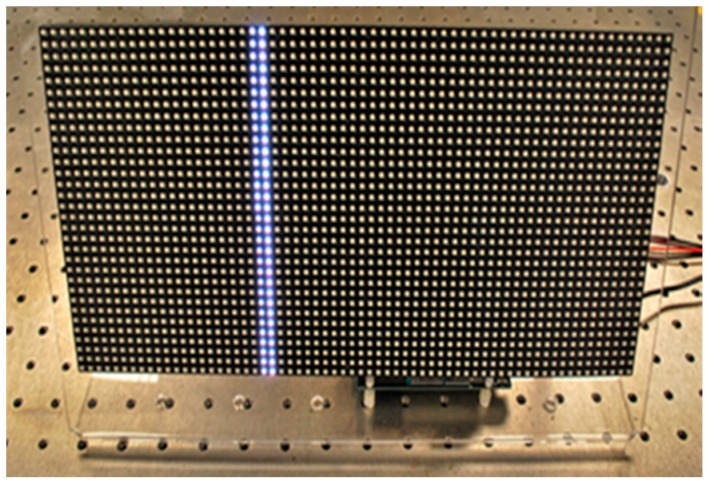
Prototype pseudo-moving target created by LED lines marching across the array.

The bioinspired sensor is well-suited to identifying displacements of moving targets; however, long duration movements, such as slippage along a fault line, are almost static. To exploit the strengths of the bioinspired sensor, a pseudo-moving target is employed so that the sensor is responding to motion in its field of view, even when the target is not moving. The prototype pseudo-moving target is created from an array of light-emitting diodes (LEDs) programmed to display a sequence of lines marching across the array (Figure 3).

**Figure 4:**
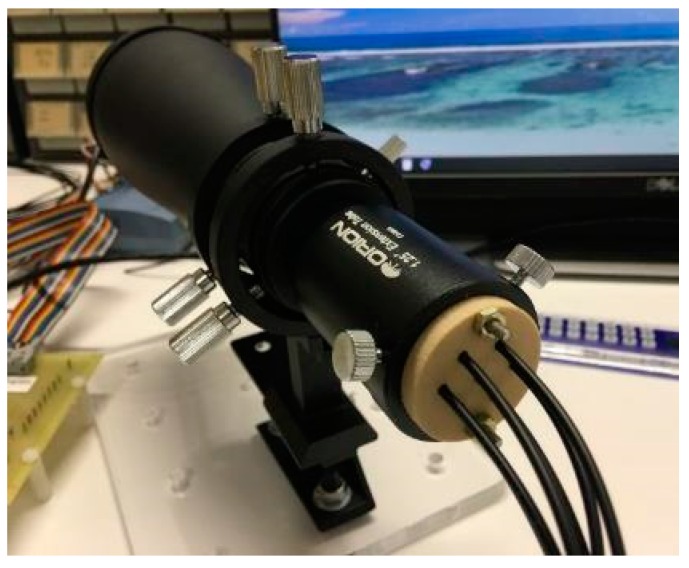
Sensor head installed in Orion 70 mm Multi-Use Finder Scope with fiber optic guides exiting the rear.

In a fault monitoring application, the sensor and target would be located on opposite sides of the fault being monitored. The sensor would be mounted on a geodetic monument built on stable ground outside of the moving fault zone with line-of-sight to the target. The target would be mounted inside the fault zone. Depending on the fault or landslide being monitored, the sensor to target distance could be as little as 6 m or as much as 100 m. External front-end optics are required for the sensor to obtain useful signals from targets at these distances. For cost considerations, primarily driven by the front-end optics, testing of the proposed system has been limited to 30 m. This enabled the use of relatively inexpensive astronomical telescopes (Figure 4) to increase the sensor’s range. The sensor head was designed at NASA Ames to fit a standard telescope interface (31.75 mm diameter eyepiece tube). Algorithms were developed to process data gathered from the sensor to determine relative displacement of the target.

## 2. Materials and Methods

### 2.1. NASA Ames Bioinspired Sensor

#### 2.1.1. Sensor Head

The studies reported in [4,5,6,7] used a sensor that was designed and built at the University of Wyoming. This sensor was constructed with 7.5 deg angles between the optical axes of each of the seven individual lens/POF pairs, resulting in a total field of view that is too large for the acquisition of the real image created by the objective lens of the telescope. Therefore, several new sensor heads were designed and built at NASA Ames (Figure 2). Since the telescope objective lens forms a real image in the focal plane inside the eyepiece tube, all lens/POF optical axes were made parallel to each other to enable all of the sensors to acquire the image. The outer diameter of the sensor head is 31.75 mm, enabling it to fit inside the telescope standard eyepiece tube. The University of Wyoming sensor head had 3 mm lenses with a distance of 2.4 mm between the lens and the POF face [4,5,6,7]. The fault application needs as much light gathering capability as possible since the target is located far from the sensor.

Constraints on the sensor head design included the inner diameter of the telescope (31.75 mm), a limited number of lens sizes, and the use of 1.0 mm POF to interface with the photodetector. Through analysis using ray-tracing studies performed at NASA Ames, it was determined that a 6.0 mm lens coupled with 1 mm POF would be optimal with a 7.0 mm cavity or air gap between the lens and the POF face to blur the image, resulting in the desired quasi-Gaussian response to stimuli. Each POF end is coupled with an aspheric lens that is 6.0 mm in diameter (replicated asphere, 6.0 mm diameter, 6 mm focal length, uncoated, P/N 68-106, Edmund Optics, Barrington, NJ, USA).

**Figure 5:**
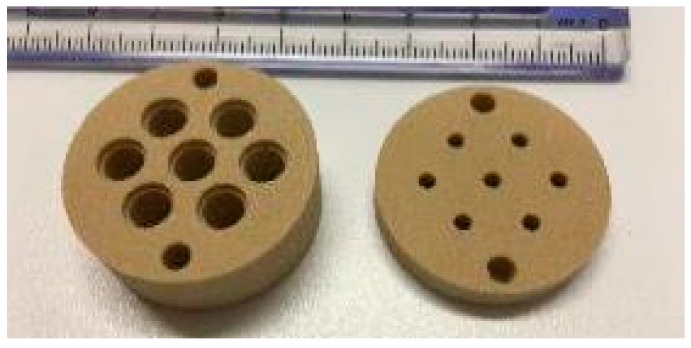
Lens side of the seven-lens sensor head (**left**), and POF side of sensor head (**right**).

**Figure 6:**
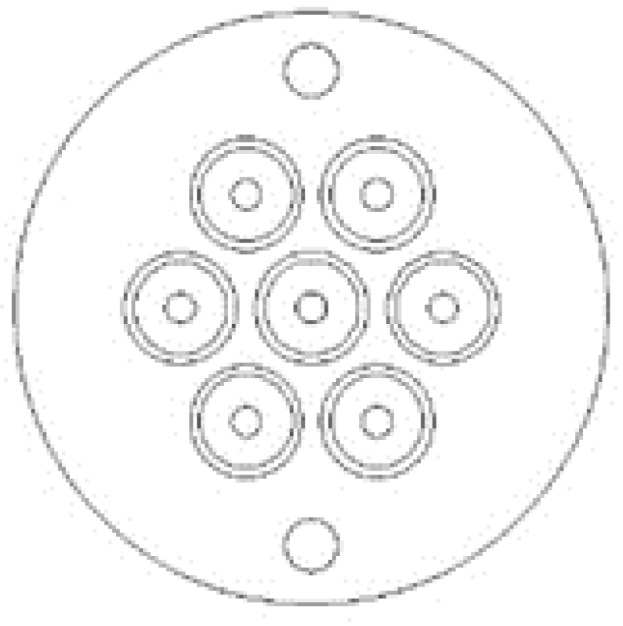
Computer-aided design drawing of the seven-lens sensor head, lens side, with optical axis pitch of 7.00 mm.

**Figure 7:**
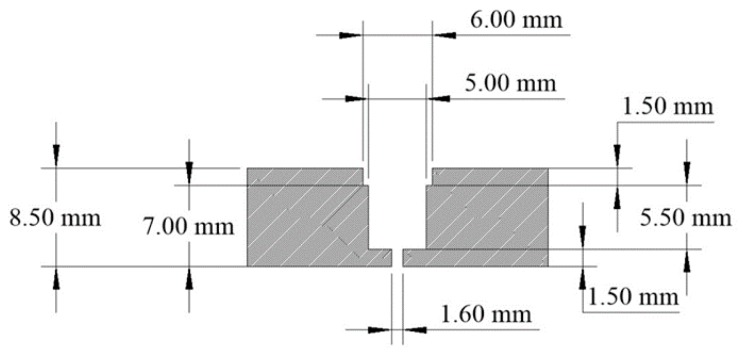
Optical cavity cross section, lens side.

**Figure 8:**
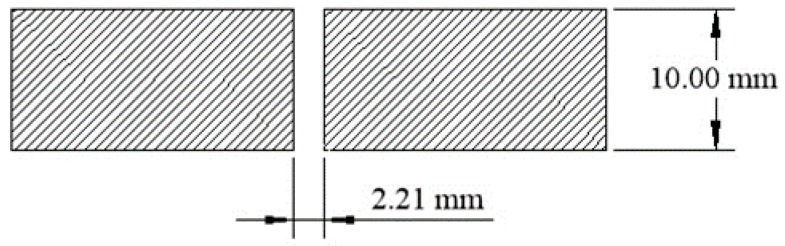
Optical cavity cross section, POF side.

The sensor head is constructed of two pieces (Figure 5). The front face, or lens side, holds the 6 mm aspheric lenses above the cavity and has a 1.6 mm opening at the bottom to allow the light to pass to the fiber core (Figure 6 and Figure 7). The rear face, or POF side, of the sensor head is designed to hold the 1.0 mm POFs (2.2 mm outer diameter with jacket) in position under the cavity below the lens (Figure 8).

#### 2.1.2. Front-End Optics

Astronomical telescopes provide a good solution for front-end optics to increase the range of the sensors since they have excellent light gathering capabilities, a long focal length, and easy access to the primary focal plane. To prove the feasibility of using astronomical telescopes for this application, a low-cost 70 mm finder scope (Orion 70 mm Multi-Use Finder Scope, $99.99, Product item #07220, Orion Telescopes and Binoculars, Watsonville, CA, USA) was initially selected for testing (Figure 4). This telescope produced good quality data for relatively short distances of approximately 6 m between the target and the telescope/sensor combination. For distances greater than 6 m, it was determined that optics with a larger aperture for increased light gathering capability and a longer focal length for larger magnification were required.

A low-cost 102 mm Maksutov-Cassegrain telescope (Orion Apex 102 mm Maksutov-Cassegrain Telescope, $229.99, Product item # 09823) was selected for tests where the target to sensor distance was above 6 m, along with a 2× Barlow lens (Orion Telescopes and Binoculars, Watsonville, CA, USA) to further increase the focal length for better magnification. Higher magnification requires more precise telescope alignment with the target, so a low-cost alt-azimuth screw mount was incorporated (Orion Precision Slow-Motion Adapter, $39.99, Product item # 07033, Orion Telescopes and Binoculars). This tripod adapter has screw-driven fine adjustments for azimuthal as well as elevation directions, approximately ±20 degrees. All tests with this telescope were performed with the telescope mounted on a camera tripod.

Figure 4 shows the NASA Ames seven-lens sensor head installed in the 70 mm finder scope. Astronomical telescopes are designed for targets at infinity. The targets used for this experiment are only 2 to 30 m from the telescope, so the location of the real image formed by the telescope objective lens is further away from the objective lens than for targets at infinity. Since the sensor head needs to be focused on the real-image, a standard eyepiece extension tube was used to position the sensor head so it was viewing the real-image.

**Figure 9:**
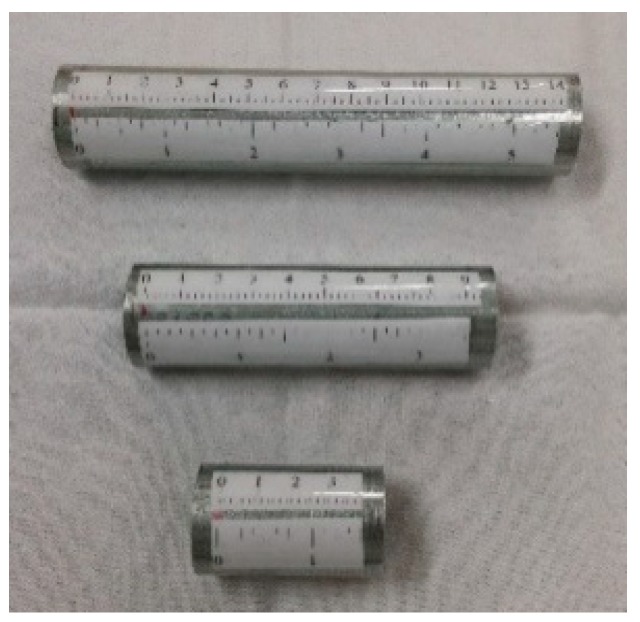
Image plane locators.

**Figure 10:**
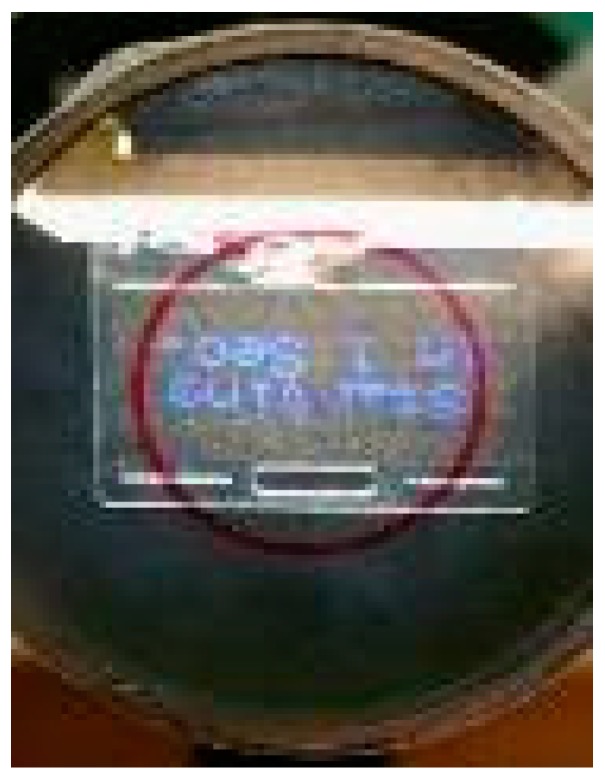
Real image of target in image plane locator.

The current telescope/sensor system has no automatic imager for adjusting the focus, i.e., a system for finding the precise location of the sensor head relative to the real image plane. Simple real image plane locators were constructed from metal tubes with a focal plane located at one end. The focal plane is a 6.35 mm acrylic plate with a matte finish on one side and a polished transparent surface on the other (Figure 9 and Figure 10).

The focusing procedure is as follows:

1. For a given target to telescope/sensor distance, find the real image focal plane visually using the real image locator tube. Note the distance between the real image plane and the end of the eyepiece extension tube using the ruler attached to the real image locator tube.
2. Insert the sensor head to position the lens side of the sensor head so it is aligned with the real image focal plane, prior to data acquisition.

#### 2.1.3. Printed Circuit Board and Data Acquisition

The PCB used for the studies presented here was designed and built by researchers at the University of Wyoming [7,9]. The PCB has seven photodiode detectors (IFD-92 phototransistor detector, Industrial Fiber Optics, Inc., Tempe, AZ, USA) housed in a fiber optic package. Each photodetector is connected to the end of one POF from the sensor head. The photodetector converts light (photons) coming through the POF into an electrical current. This current is fed to a logarithmic amplification circuit that amplifies and converts the signal to an analog voltage using a now obsolete TI LOG102 amplifier (Texas Instruments, Inc., Dallas, TX, USA). The signal is then passed through two active filters. A notch filter centered at 60 Hz is used to remove noise due to interior lighting. Since the signal of interest is in the near-direct current (DC) range, the second filter is a fourth-order Butterworth low-pass filter with a 50 Hz cutoff frequency. To accommodate different environmental conditions, such as fog, photodetectors that are sensitive in the infrared (IR) range could be used.

The seven channels of analog output coming from the sensor’s PCB were connected to a data acquisition system, USB-2408-2AO (Measurement Computing Corp., Norton, MA, USA). This is a universal serial bus (USB)-based data acquisition system capable of 8 channel differential-inputs, or 16 channel single-ended inputs. The digitization resolution is up to 24 bits. The system was configured as seven channel single-ended inputs with a 16-bit resolution. All data was taken at 100 Hz per channel per second. Commercial off-the-shelf software, DAQami (Measurement Computing Corp.), was used for all data acquisition. When looking at the rear of the sensor when it is facing the target, the channels are labeled as follows: 1—top row left; 2—top row right; 3—middle row left; 4—middle row center; 5—middle row right; 6—bottom row left; and 7—bottom row right.

#### 2.1.4. Pseudo-Moving Target

The prototype pseudo-moving LED target (Figure 3) is constructed from an Adafruit 64 × 32 RGB LED Matrix, Product ID: 2277 (Adafruit, New York City, NY, USA). The individually addressable, 12-bit RGB LEDs (4-bits per red, green, and blue) are installed on a panel, at 5 mm pitch (spacing between LEDs), with 64 LEDs in the *x*-direction and 32 in the *y*-direction. The size of the panel is 318 mm × 158 mm × 15 mm. An LED monitor could be used for this application; however, its cost and power requirements are higher than the selected LED target and its microcontroller. While a LED monitor could give a higher resolution, the 5 mm pitch was sufficient for the fault monitoring application.

Software was developed to control the illumination of the target LEDs. The target has an initial illumination pattern that is displayed on startup, enabling the software that interprets the sensor data to recognize that the target is commencing operation. The initial pattern remains lit for 1.0 s, after which all of the LEDs are turned off for 0.5 s. Next, starting on the left side of the panel, one vertical line of LEDs is illuminated for 0.5 s, then all of the LEDs are turned off and the next line to the right is illuminated. Once each of the 64 columns of LEDs have been illuminated, all of the LEDs are turned off for 0.0025 s, after which the lines march across the display again. The lines march across the LED display five times, after which the LEDs are all turned off. These configuration parameters can be easily changed through a software interface.

### 2.2. Fault Movement Estimation

**Figure 11:**
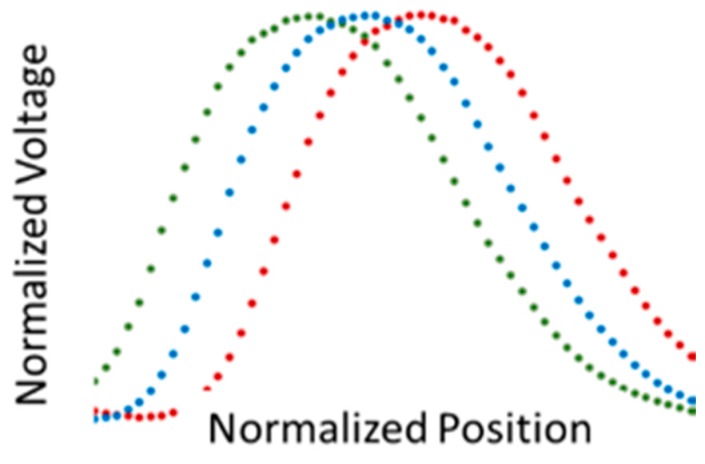
Calculated quasi-Gaussians for a target located −2 (green), 0 (blue), and 2 cm (red) displacement from the centerline of the sensor.

The approach to estimating fault movement involves comparisons of quasi-Gaussian distributions from the individual sensors’ responses to a sequence of vertical illuminated LED lines moving from left to right across the LED target (Figure 11). Each point in the quasi-Gaussian represents the sensor’s response to illumination of a single LED line. Displacement of the LED array to the left (right) results in translation of the quasi-Gaussian to the right (left). The sensor will detect the displacement of the target, not the actual motion, during the fault slip event. The target displacement can be calculated from the translation of the quasi-Gaussian to the left or right and the LED spacing. Note that the actual positions of the optical axes of the sensors comprising the sensor head are not used in the calculations. These positions are static after installation of the sensor. Only the relative changes in the quasi-Gaussian are being measured.

#### 2.2.1. Identification of the Quasi-Gaussian

**Figure 12:**
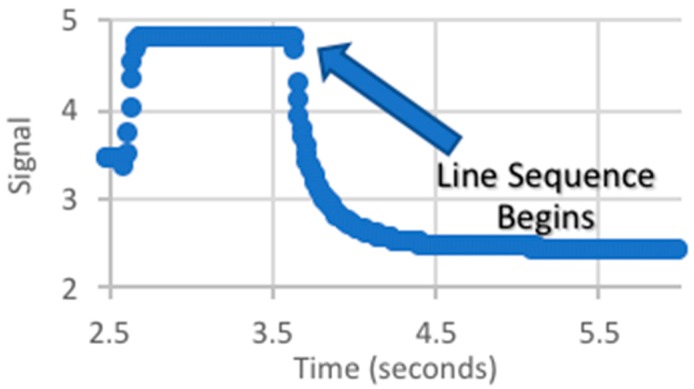
Signal (V) at the beginning of line sequence.

Prior to identifying the quasi-Gaussian from an individual sensor, the sensor data must be analyzed to determine the start of the illumination sequence and the length of time each LED line was illuminated. There was no link between the target and data acquisition system that specified when the sequence began. Therefore, a unique signal behavior was used to identify the start of the sequence. This was achieved by illuminating a significant number of LEDs for a period of time, resulting in a plateau region at the signal’s maximum. When the LED line sequence began, there was a sudden drop in the signal, which was identified with the beginning of the sequence (Figure 12).

**Figure 13:**
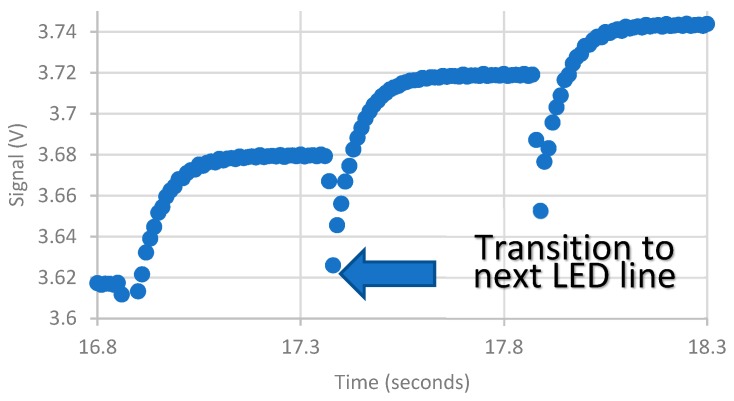
Signal at transition to next LED line.

To identify the transition between LED lines, the target was programmed to illuminate a line for a nominal period of time; all LEDs were then turned off, and the next line was then illuminated. This resulted in a sudden drop and then rise in the signal (Figure 13). Using these sudden variations, the total time that the target was dark and then a single LED line illuminated could be estimated. Once this time was identified, LED line numbers could be associated with data segments.

When developing the pseudo-moving target methodology for fault monitoring, it was assumed that the rises/falls between plateaus would be short, and that the plateau regions would be quickly established. Due to the smoothing seen in the data, possibly inherent to the data acquisition system, the rise/fall time was longer than anticipated and was amplitude-dependent—the stronger the signal, the shorter the time to reach a plateau.

**Figure 14:**
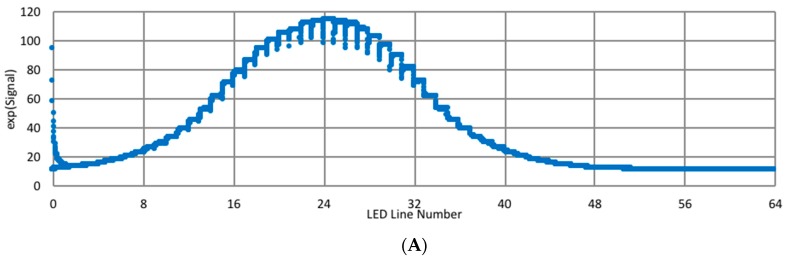

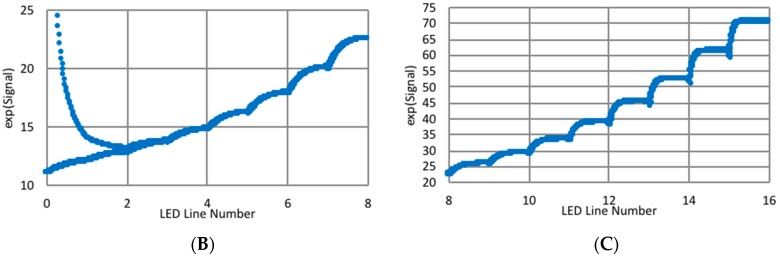
Sample signals (V) for LED lines from (**A**) one entire pass of pseudo-moving target, (**B**) LED lines 0–8, and (**C**) LED lines 8–16.

To increase the likelihood of reaching a plateau, the time each LED line was displayed for was increased to 0.5 s. This translated to 32 s for the entire target sequence; any longer was thought to be untenable for the application. A representative signal for 3.5 cycles of the LED target is shown in Figure 14 plotted against the LED line number. Even with the relatively long display time, plateaus were not achieved, particularly when the signal was weaker.

**Figure 15:**
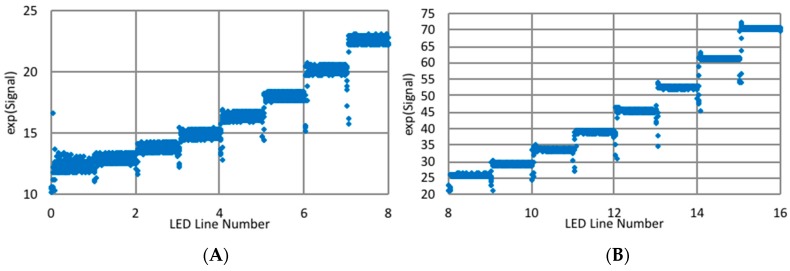
Signal (V) with corrections for rise/fall. (**A**) LED lines 0–8, and (**B**) LED lines 8–16.

**Figure 16:**
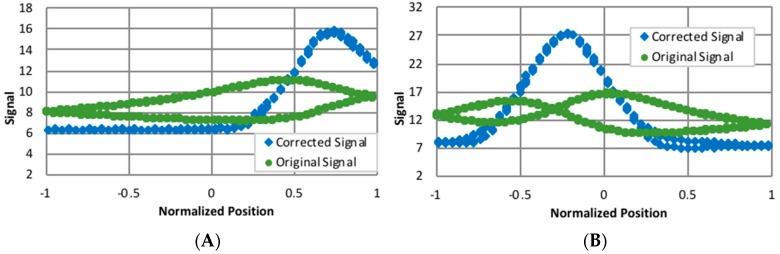
Uncorrected and corrected signals (V) for a laterally moving paper target for (**A**) channel 2 and (**B**) channel 3 of the sensor.

Using several data sets, the rise and falls were modeled, and corrections to the signal were identified. Examples of corrected data are shown in Figure 15. To verify that the long rise/fall times were due to the acquisition system and not the target, the corrections have been applied to a paper target moving laterally in front of the target (Figure 16). The corrections transformed signals exhibiting severe distortions and significant hysteresis as the target moved up and down into functions more closely resembling Gaussians. Unfortunately, these corrections are large and preliminary data indicate they are gain-dependent. Also, corrections for the data smoothing were not incorporated into the algorithms for estimating fault movements. Therefore, only a segment of the available data was averaged to provide the response to the illumination of an LED line. In general, the data was obtained during the end of the exposure time when a plateau was nearly attained.

#### 2.2.2. Displacement Estimates

Target displacement is obtained by determining the relative offsets of the quasi-Gaussian for a target displaced relative to that of a reference target. Several factors in identifying the offset, for example, background lighting which impacts the signal’s peak to background ratio, were considered when determining the relative offsets.

Each quasi-Gaussian is represented by 64 points, the averaged responses to the illumination of 64 LED lines. The deviation from a true Gaussian is not negligible; therefore, when calculating the offsets, look-up tables instead of math models of the quasi-Gaussians were used. To identify the displacement, the reference quasi-Gaussian was translated to the left or right and, if desired, the width of the quasi-Gaussian was varied. Second-order interpolation was used to map the translated and stretched/compressed Gaussian to 64 regularly spaced points correlating with those of the displaced target’s quasi-Gaussian. To provide the best fit of the reference to the displaced target’s quasi-Gaussian, a least-squares analysis was used to modify the background and peak values of the reference, and the root mean square (RMS) difference between the modified reference and displaced quasi-Gaussian was calculated. Using a searching routine, this process was repeated, and the target displacement was identified with the fit with the minimum RMS difference.

**Figure 17:**
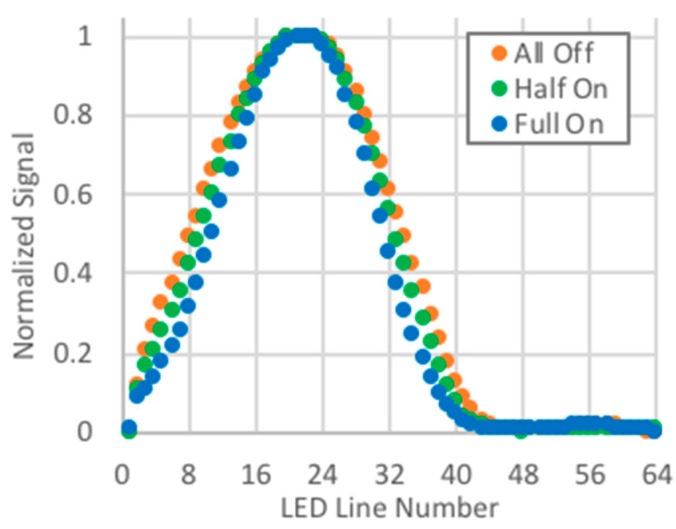
Normalized signal.

**Figure 18:**
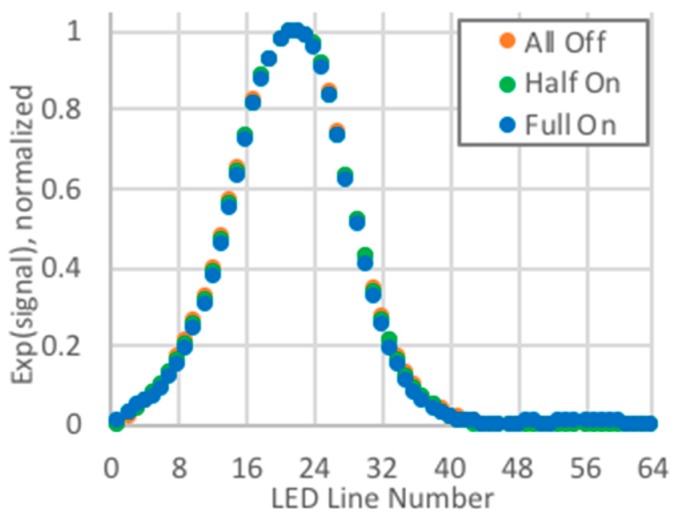
Exponential of signal, normalized.

Initially, unmodified signals were used for identifying the displacement. When base levels were removed from signals acquired during indoor tests with the overhead lights fully on, half on, and all off, and the signals were then normalized, the signal width had a definite dependency on the background lighting (Figure 17). Therefore, when the reference and displaced quasi-Gaussians were obtained with different background lighting, the capability of changing the width of the quasi-Gaussian was a requisite for obtaining good displacement estimates. The PCB used logarithmic compression when amplifying the signals, and when the exponential of the signal was taken, this dependency on the background lighting was almost eliminated, and good estimates were obtained without any modifications to the width of the reference quasi-Gaussian (Figure 18).

### 2.3. Analysis Program

**Figure 19:**
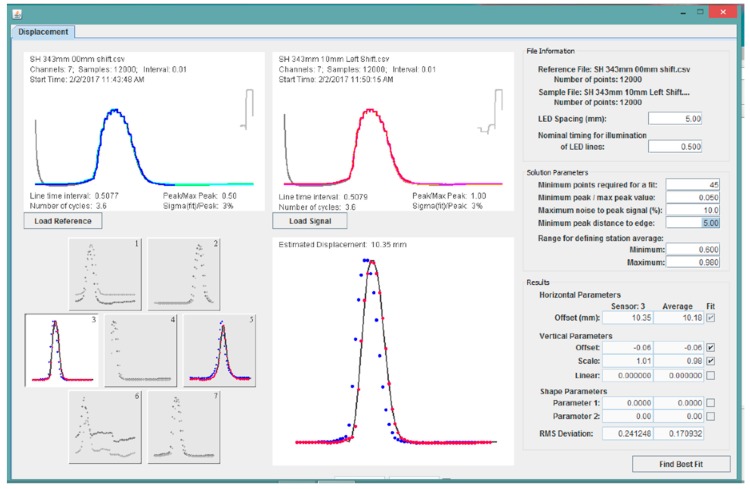
Graphical user interface.

User-friendly software was developed for analysis of the sensor data. A graphical user interface (GUI) provides visual verification of the various steps in the analysis process (Figure 19), particularly the identification of the quasi-Gaussian for the reference and sample (displaced) signals (two upper plots), and the fit of the translated reference quasi-Gaussian to the displaced quasi-Gaussian (lower plots). Included in the upper plots is information regarding the data file, including the name of the file, the number of channels, the number of cycles of the LED target recorded, the period associated with the illumination of each line, and the RMS variation in the signal from cycle to cycle. There are up to seven channels of data, one for each individual sensor, and the user can easily highlight a channel by selecting any of the images in the lower left of the interface.

In the upper right of the interface are the user-specified options for identifying the quasi-Gaussians (Table 1). In the lower right corner are options and results for the fits of the reference signal to the displaced signal (Table 2). For each item, the value for the displayed channel (individual sensor) and the average for all channels are displayed.

## 3. Results and Discussion

Three sets of tests were performed: (1) indoor tests with no front-end optics; (2) outdoor tests with an Orion 70 mm Multi-Use Finder Scope (70 mm optical diameter, 279 mm focal length); and (3) outdoor tests with an Orion Apex 102 mm Maksutov-Cassegrain Telescope (102 mm aperture, 1300 mm focal length).

### 3.1. Indoor Tests

Indoor tests were conducted with the interior overhead lights fully on, half on, and all off. The distance between the sensor head and the target was 35 mm, and the target was placed at three locations: the centerline, 10 mm to the left of the centerline, and 20 mm left of the centerline. Approximately 3.5 cycles of pseudo-motion were captured. The estimated displacements for each channel (individual sensor) and the averaged displacement are given in Table 3; the averaged value excludes channel 7. The data obtained with channel 7 was nosier, and the displacements estimated using the channel 7 signals were often outliers. With the exclusion of channel 7, the displacements estimated from the individual sensors are within 0.12 mm of the average, and the averages are within 0.25 mm of the nominal displacement. It should be noted that the nominal displacement is not exact and that the differences between the estimated and nominal displacement are not true measures of the uncertainties. A better estimate of the error may be obtained by comparing the displacement (19.91 and 19.88) calculated for the reference at 0 mm and the sample at 20 mm with the sum (19.86 and 19.91 mm) of the displacements for the reference at 0 mm and sample at 10 mm (9.75 and 9.80 mm) and the reference at 10 mm and the sample at 20 mm (10.11 mm). The max difference between these two sets of estimates is 0.05 mm, and it is therefore not unreasonable to expect uncertainty in the displacement estimates in the order 0.1 mm for a controlled environment. This is significantly less than the LED line spacing of 5 mm.

### 3.2. Outdoor Tests at 6 m

**Figure 20:**
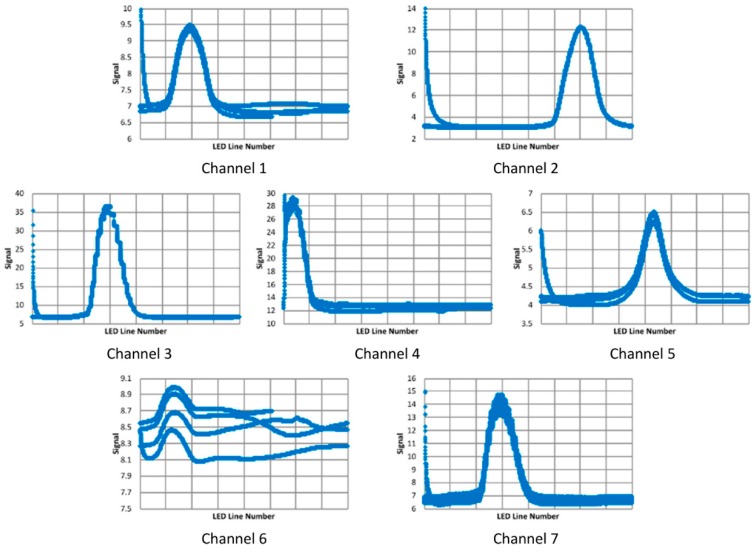
Quasi-Gaussian response signals (V) for each LED line (1–64) for the seven sensor channels for outdoor tests with the Orion 70 mm Multi-Use Finder Scope.

Outdoor tests were performed with the target at 6 m and the Orion 70 mm Multi-Use Finder Scope as the front-end optics. Data was collected with the distance between the front-end lens and the sensor of 343 mm and with the target at two locations, a reference location and one with the target offset to the left by, nominally, 10 mm. The responses for the reference location are shown in Figure 20. Note that 3.6 cycles of pseudo-motion are overlaid in these plots. With channels 1, 4, 5, and 7, there are definite issues with repeatability, and with channel 6, there is a significant deviation from a quasi-Gaussian response.

When identifying the displacement, constraints were placed on the data: a maximum was placed on the noise to peak ratio (20%), and weak signals (channels with a peak value that was less than 5% of the maximum peak for all seven channels) and signals with peaks too close to the edge were discarded. The estimated target displacements, channel 1: 10.49, channel 2: 10.58, channel 3: 10.35, channel 4: N/A, channel 5: 10.0, channel 6: N/A, and channel 7: 10.32 mm, with an average of 10.35 mm, are surprisingly consistent, although the displacement for channel 5, with its fairly weak signal, appears to be smaller than that for the other channels. Again, it should be noted that the nominal displacement was approximate, and therefore no conclusions can be drawn regarding the accuracy of the displacement estimates; the consistency of the measurements would suggest uncertainties are in the order of a few tenths of a millimeter.

### 3.3. Outdoor Tests at 30 m

**Figure 21:**
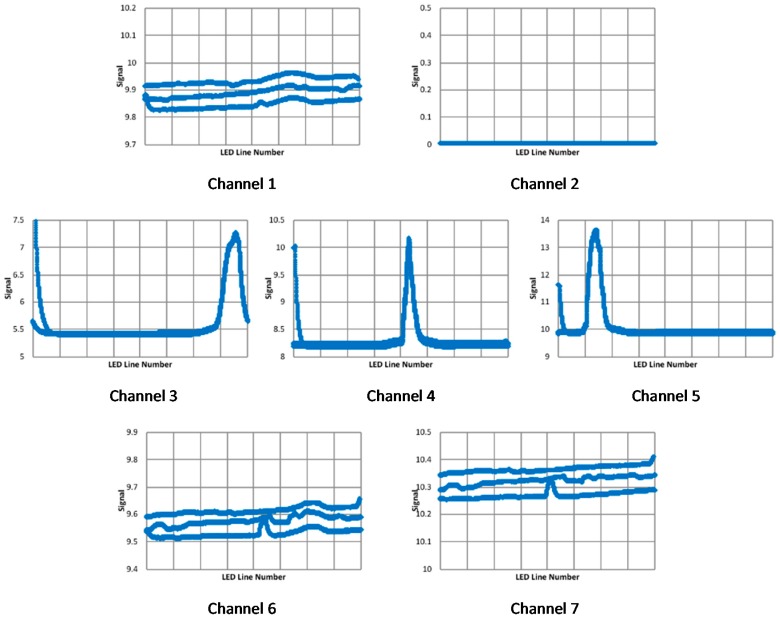
Quasi-Gaussian response signals (V) for each LED line (1–64) for the seven sensor channels for outdoor tests with Orion Apex 102 mm Maksutov-Cassegrain Telescope.

**Figure 22:**
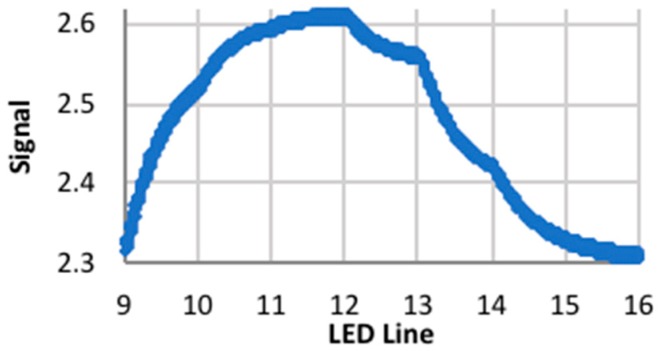
Peak signal (V) at 30 m—channel 5.

Outdoor tests were performed with the target at 30 m, and the Orion Apex 102 mm Maksutov-Cassegrain Telescope and a 2× Barlow lens as the front-end optics. At this distance, and with the specified front-end optics, the target image was not in view of all of the individual sensors, and only three of the seven channels exhibited quasi-Gaussian behavior (Figure 21). The target was placed at five locations, including on the centerline, and 10 and 20 mm to the left and right of the centerline. For this configuration, the signals were fairly weak and in the amplitude region where there was significant smoothing of the signal (Figure 22). The signal was either monotonically increasing or decreasing, and there were no sudden falls followed by sudden rises that could be identified with turning the LED array off and then illuminating the next line in the sequence. Therefore, the exposure time was fixed to an appropriate value.

Estimated target displacements are shown in Table 4. Two issues are immediately apparent. First, for the three channels, the scatter in the measured displacements is larger than that at the shorter distances. A major contributor to the increase in the scatter is the peaks are narrower; therefore, there are fewer points defining the quasi-Gaussian, and the accuracy of the interpolated values for the translated reference quasi-Gaussian has larger uncertainties.

The second issue is that the average displacement differs by a few millimeters, not a few tenths of a millimeter, from the nominal value. The start time of the LED line sequence is initially identified with the sudden reduction in amplitude. The rapid falls and rises associated with turning off the LED array and then progressing to the next LED line are used to adjust the start time. Since the smoothing of the data generally removed these falls and rises, the adjustments were not accurate.

Using a visual inspection of the signals, changes in the signal associated with progression to the next LED line were used to adjust the start time for the target sequence. This was done for two of the data sets: (1) the target on the centerline; and (2) the target shifted to the right by 10 mm. With these corrections, the displacement estimates are within 0.8 mm of the nominal displacement (Table 5).

## 4. Conclusions

Identifying appropriate sites for landing a spacecraft or building permanent structures is critical for the exploration of planetary bodies, including asteroids, moons, and planets. By tracking the movement of land masses or structures on a planetary surface, scientists can better predict issues that could affect the integrity of the site or structures. Scientists will need new lightweight creep meters for planetary missions to monitor land movement for structures located near faults to assess the hazard of the fault. Additionally, scientists and engineers will need to monitor structures constructed on planetary surfaces for movement that could signify support or structural issues. In this work, we focused on the application of monitoring the movement of faults, although the sensor and algorithms presented here could be used to remotely measure the displacement of many other objects of interest for the exploration of planetary bodies.

A new bioinspired optical sensor head with front-end optics designed and built at NASA Ames was described, along with its use for remotely tracking targets that are 1–60 m from the sensor and move small displacements (1–10 mm). A pseudo-moving target having sequential illumination of lines in an LED array was used to generate signals that mimic those of a moving target. Algorithms were developed that relate these signals to quasi-Gaussians and identify the displacement by comparing the quasi-Gaussians for a reference with the displaced target. This process was tested for three distances between the sensor and the target: 0.35, 6, and 30 m. Front-end optics in the form of telescopes were used to magnify the target image for the tests at 6 and 30 m. For the 0.35 m tests, estimated uncertainties were found to be in the order of a tenth of a millimeter. At 6 m, the estimated error was in the order of a few tenths of a millimeter. For the target at 30 m, difficulties were introduced by the extreme smoothing of the signal and the narrowness of the quasi-Gaussian peak. However, accuracies in the order of 1 mm or less were obtained when corrections were made to the estimated duration for the line illumination and to the start of the target sequence. These difficulties could be alleviated by broadening the quasi-Gaussian peak, possibly by moving the sensor farther away from the image plane, and reducing the smoothing observed in the acquired data. Future work will address the signal plateau and data smoothing issues, in addition to the problems encountered at longer distances related to the signal strength. Work has started on designing and building a new PCB with an improved amplification circuit and on-board analog to digital conversion.

## Figures and Tables

**Table 1:** User-specified options for GUI.

Option	Purpose
LED spacing (mm)	Information to translate LED line number to distance
Nominal timing for illumination of LED lines	Starting value for timing estimates
Minimum points required for a fit	Constrains solutions
Minimum peak/maximum peak ratio	Identify and discard weaker signals
Maximum noise to peak signal (%)	Identify and discard noisier signals
Minimum peak distance to edge	Discard signals where the quasi-Gaussian is not fully captured
Range for defining station average (minimum and maximum)	Define segment of signal to be identified with the plateau region

**Table 2:** Options for fit of reference signal to displaced signal.

Item	Purpose
Offset (mm)	Estimated displacement
Vertical Parameters (user can turn on/off)	
Offset	Adds a constant to the signal
Scale	Modifies the peak to background value
Linear	Adds a linear variation when modifying the peak to background value
Shape Parameters (user can turn on/off)	
Parameter 1	Parameters used to change the shape of the quasi-Gaussian during the fitting process
Parameter 2	
RMS Deviation	RMS for the fit of the reference quasi-Gaussian to the sample quasi-Gaussian

**Table 3:** Displacement estimates for indoor test with no front-end optics.

Reference File		Sample File		Nominal Offset (mm)	Estimated Displacement (mm)								
Overhead Lights	Shift (mm)	Overhead Lights	Shift (mm)		Channel							Mean	SD
					1	2	3	4	5	6	7		
Full On	0	Half On	0	0	0.00	0.03	0.00	0.00	0.02	0.00	0.13	0.01	0.01
Full On	0	All Off	0	0	0.00	0.07	0.00	0.00	0.06	0.00	0.54	0.02	0.03
Half On	0	Full On	10	10	9.79	9.85	9.82	9.62	9.72	9.68	10.05	9.75	0.09
Full On	0	Half On	10	10	9.88	9.92	9.88	9.67	9.74	9.72	10.18	9.80	0.10
Half On	0	Half On	20	20	19.94	20.00	20.00	19.78	19.88	19.86	10.20	19.91	0.09
Full On	0	Half On	20	20	19.85	20.00	20.00	19.73	19.86	19.82	20.07	19.88	0.11
Half On	10	Half On	20	10	10.00	10.14	10.15	10.09	10.14	10.13	10.00	10.11	0.06

**Table 4:** Displacement estimates for outdoor test using an Orion Apex 102 mm Maksutov-Cassegrain Telescope.

Reference File Offset (mm)	Sample File Offset	Nominal Offset (mm)	Estimated Displacement (mm)				
			Channel			Mean	SD
			1	2	3		
0	−20	−20	−24.10	−25.00	−23.99	−24.36	0.55
0	−10	−10	−12.76	−12.21	−12.29	−12.42	0.30
0	10	10	14.62	14.52	14.45	14.53	0.09
0	20	20	18.11	17.90	17.32	17.77	0.41

**Table 5:** Displacement estimates for outdoor test using an Orion Apex 102 mm Maksutov-Cassegrain Telescope with adjusted start time.

Reference File Offset (mm)	Sample File Offset	Nominal Offset (mm)	Estimated Displacement (mm)				
			Channel			Mean	SD
			1	2	3		
0	−20	−20	−20.31	−20.48	−20.30	−20.37	0.10
0	−10	−10	−10.00	−10.00	−10.00	−10.00	0.00
0	10	10	9.88	10.00	9.82	9.90	0.09
0	20	20	20.80	20.00	20.00	20.27	0.46
